# How to Manage Small Intestine (Jejunal and Ileal) Neuroendocrine Neoplasms Presenting with Liver Metastases?

**DOI:** 10.1007/s11912-021-01074-2

**Published:** 2021-05-20

**Authors:** Bruno Niederle, Andreas Selberherr, Martin B. Niederle

**Affiliations:** 1 Department of General Surgery, Divison of Visceral Surgery, Medical University of Vienna, Waehringer Gürtel 18-20, A-1090 Vienna, Austria; 2 Department of Anaesthesia, Intensive Care Medicine and Pain Medicine, Medical University of Vienna, Waehringer Gürtel 18-20, A-1090 Vienna, Austria

**Keywords:** Neuroendocrine tumour (neoplasm), Small bowel, Jejunum, Ileum, Locoregional surgery, Stage IV

## Abstract

**Purpose of Review:**

Small intestinal neuroendocrine neoplasms (siNENs) are slowly growing tumours with a low malignant potential. However, more than half of the patients present with distant metastases (stage IV) and nearly all with locoregional lymph node (LN) metastases at the time of surgery. The value of locoregional treatment is discussed controversially.

**Recent Findings:**

In stage I to III disease, locoregional surgery was currently shown to be curative prolonging survival. In stage IV disease, surgery may prolong survival in selected patients with the chance to cure locoregional disease besides radical/debulking liver surgery. It may improve the quality of life and may prevent severe local complications resulting in a state of chronic malnutrition and severe intestinal ischaemia or bowel obstruction.

**Summary:**

Locoregional tumour resection offers the opportunity to be curative or to focus therapeutically on liver metastasis, facilitating various other therapeutic modalities. Risks and benefits of the surgical intervention need to be balanced individually.

## Introduction

Neuroendocrine cells, distributed throughout the body and also diffusely scattered in the gastrointestinal (GI) tract, may give rise to neuroendocrine neoplasms (NENs). As demonstrated in a prospective, 1-year incidence study, 44 out of 306 (14.4%) newly diagnosed gastro-entero-pancreatic NENs of the GI tract were located in the small intestine [1•]. Beside locations in the stomach (23%) and appendix (21%), the small bowel was seen to be the third most common site with an incidence of 0.29 per 100,000 inhabitants [1•].

Small intestinal NENs (SiNENs) are the most common type of small bowel malignancy and are diagnosed in 38 to 52% of all malignant tumours of the small bowel [1•, 2].

## Background

By definition, all NENs generally are malignant lesions, regardless of their location [3]. Following the oncologic principles, their malignant behaviour corresponds to the grading and TNM staging of the primary tumour at the time of diagnosis [4].

Although the majority of primary tumours were graded G1 (37%) and G2 (63%) [1•] and are therefore slow-growing lesions with an overall good prognosis, only a minority of patients presented with “localised disease” (WHO stage I/II; European Neuroendocrine Tumour Society [ENETS] I to IIIa: N0, M0 [16.1%]) at the time of diagnosis. The majority was documented with lymph node (LN) metastasis (“regionalised” disease; WHO stage III; ENETS stage IIIb; N1, M0; [35.5%]) or with “distant” metastasis (distant disease; WHO/ENETS stage IV, M1; [54.8%]—Tables 1 and 2) [1•].

**Table 1:** TNM classification of small intestinal (si) neuroendocrine neoplasia (NEN)

T - Primary tumour	
x	Primary tumour cannot be assessed
0	No evidence of primary tumour
1	Tumour invades lamina propria or submucosa, size ≤ 1 cm
2	Tumour invades muscularis propria or size > 1 cm
3	Tumour invades subserosa without perforation of the serosa
4	Tumour perforates the visceral peritoneum (serosa) or invades other organs/neighbouring structures For any T, add (m) for multiple tumours
N - Regional lymph node metastasis	
x	Regional lymph nodes cannot be assessed
0	No regional lymph node metastasis
1	Less than 12 regional lymph node metastases without mesenteric mass(es) greater than 20 mm in size
2	12 or more regional lymph nodes and/or more regional nodes and/or mesenteric mass(es) greater than 20 mm in maximum dimension
M - Distant metastasis	
x	Distant metastasis cannot be assessed
0	No distant metastasis
1	Distant metastasis
	a Hepatic metastasis only
	b Extrahepatic metastasis only
	c Hepatic and extrahepatic metastases

**Table 2:** WHO 2019 and ENETS 2007 staging of siNEN and frequency of locoregional and distant disease [1•, 44•, 75••, 76••]

WHO			ENETS			ENETS+WHO	*n* (%)	Disease
Stage	T	N	Stage	T	N	M		
			0	Tis	0	0	0	Local
I	1	0	I	1	0	0	2 (6.5)	Local
II	2	0	IIa	2	0	0	1 (3.2)	Local
II	3	0	IIb	3	0	0	2 (6.5)	Local
III	4	any N	IIIa	4	0	0	0	Local
III	any T	Any N	IIIb	any T	1	0	11 (35.5)	Regional
IV	any T	any N	IV	any T	any N	M1 a-c	15 (48.4)	Distant

In a single-centre study, LN metastasis occurred in up to 93%, and liver metastasis was revealed in 61% of the patients [5••]. Therefore, in more than half of the patients with siNENs, the treatment concepts are to focus not only on the primary tumour and its LN metastasis but also more intensively on distant metastasis, tumour-related symptoms and carcinoid heart disease, if present. Individualised multimodal treatment is to be discussed in interdisciplinary tumour boards to prolong survival [6]. Structured diagnostic and therapeutic algorithms are thus mandatory to select the individualised therapy with the highest chance to improve prognosis and quality of life (Tables 3 and 4).

**Table 3:**
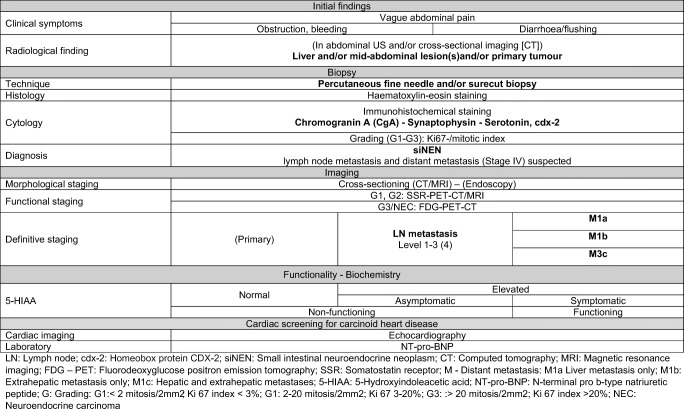
Diagnostic pathway in siNEN

**Table 4:** Therapeutic pathways in siNEN Stage IV (pT1-4; pN1/2)

	M 1a	M1a-c	M1a-c
Grading	G1-2	G1-2	G3/NEC
Surgical treatment	Radical resection curative intent	Palliative resection	NO resection
Surgical technique	Local radical open	Local radical open	
T	Primary tumour(s)	Primary tumour(s)	Due to Local ([T], N) inoperability
N	LN dissection	LN dissection	
	Levels 1–3 (4)	Levels 1–3 (4)	
M	Typical/atypical Liver resection	Liver debulking - Resection of PC	
Co-morbidity	no	no	Yes/no
Aim	Free from tumour	To avoid local complications by the tumour/LN metastasis (obstruction, bleeding, ischaemia)	Palliation
		To improve: Quality of life Prognosis?	

*LN* Lymph node; *T* Tumour, *N* Node; *M* - *Distant metastasis* M1a Liver metastasis only; *M1b* Extrahepatic metastasis only; *M1c* Hepatic and extrahepatic metastases; *PC* Peritoneal carcinomatosis; *G* Grading: *G1*< 2 mitosis/2mm^2^; Ki 67 index < 3%; *G1* 2-20 mitosis/2mm^2^; Ki 67 3-20%; *G3* > 20 mitosis/2mm^2^; Ki 67 index >20%; *NEC* Neuroendocrine carcinoma

## Aim

This article deals with the treatment of the primary tumour and LN metastasis (locoregional disease) in patients with distant metastasis on the basis of international guidelines [7•, 8•, 9•, 10•, 11•, 12•] and recently published meta-analyses [13••, 14••, 15••, 16••].

The current treatment concepts for resectable and non-resectable distant metastasis (peritoneal carcinomatosis [PC], liver) after successful (if possible, radical) treatment of locoregional disease are not discussed in detail and are summarised elsewhere [17, 18, 19].

## Functionality

SiNENs are non-functioning or functioning. The assessment of hormonal function is an important step in the diagnosis of the diseases and is induced when siNENs are suspected (Table 3). SiNENs secrete serotonin, and 5-hydroxyindoleacetic acid (5-HIAA), a metabolite of serotonin, is excreted in the urine. Determining 5-HIAA levels in the 24-h urine collection is the most useful test in confirming the diagnosis of siNENs and “carcinoid syndrome”. 5-HIAA values were significantly higher in patients with siNENs graded G2 and in patients with 25% of liver volume involvement [20•, 21•].

In the majority of patients, functionality correlates with more advanced disease. It is found in up to 45% of patients and may be caused by LN and/or distant metastasis [22]. Serotonin and other cytokines released from the tumour cells may induce fibrosis, leading to carcinoid heart disease and abdominal fibrotic reactions (desmoplasia) [23•, 24].

The symptoms of hormone hypersecretion may be mild or severe, resulting in carcinoid crisis and carcinoid heart disease. Carcinoid heart disease characterised by plaque-like deposits of connective tissue cells leading to tricuspid valve dysfunction or even heart failure may influence patients’ general health condition and may thus reduce the chances of extended surgery [25]. Carcinoid heart disease is to be excluded before deciding on treatment. Such therapy is challenging, warrants a multidisciplinary approach and can be medical or surgical, depending on the cardiac manifestations [26•].

If the diagnosis of siNENs is confirmed, all patients should routinely be put on long-acting somatostatin analogues (SSAs), which are antisecretory (and in long-term treatment, antiproliferative), in order to prevent an escalation of clinical symptoms during diagnostic work-up and subsequent treatment [27•].

### Symptoms and pathway of diagnosis

The majority of siNENs are diagnosed incidentally when unspecific upper abdominal pain (33%) or intestinal bleeding (15%) leads to further examinations [28•].

More specific symptoms for siNENs may be diarrhoea (21%) or flushing (9%) as signs of serotonin overproduction. Partial (18%) or total bowel obstruction of both functioning and non-functioning tumours may require emergent operative interventions [29]. However, all these symptoms are unspecific and may also be found in NENs located in other parts of the GI tract [28•].

Diffuse upper abdominal pain frequently corresponds to liver metastasis and is described in 20 to 67% of patients with various GI NENs when abdominal ultrasound (US) or computed tomography (CT) is initially performed for tumour “screening” (Table 3) [1•].

In some of these stage IV tumours, the type and location of the primary tumour may remain unknown even after extended, additionally performed cross-sectional imaging (e.g. MRI) or endoscopy of the upper and lower GI tract as the second and third steps of the investigation pathway.

As shown recently [30], the site of the primary NEN may be based on biopsy specimens of the liver applying a specific set of immunohistochemical markers. Positive staining for CDX2 was documented in 43 (86%) and for serotonin in 45 (90%) of 50 siNENs.

Liver biopsy not only confirms the NEN itself and helps to identify the tumour site of origin but also facilitates tumour grading (Table 3). Liver lesions are often of a higher grade than the primary or its local or regional LN metastasis [31].

### Characteristics of siNENs

#### Primary tumours

The majority of primary tumours are slowly growing submucosal lesions, only a few millimetres in size (60% ≤ 20 mm), which invade the lamina propria or submucosa or the muscularis propria and are classified as pT1 or pT2 (Table 1). The lesions are more often located in the ileum (76%) than in the distal jejunum (24%) and may be multiple in up to 54% of cases [32, 33, 34•, 35].

The verification of the primary in the small intestine is difficult and sometimes impossible with conventional radiological methods because of the per se invisible part of the bowel.

In more detailed investigations, contrast intestinal radiography, video capsule endoscopy, or double-balloon enteroscopy may be applied providing more information on the location and the number of lesions within the ileum and/or jejunum [36, 37].

CT enteroclysis appears to be inferior to video capsule enteroscopy (sensitivity and specificity: 50 and 25% vs. 38 and 100%, respectively) [38].

However, the latter endoscopic diagnostic modalities are not widely available. They may be effective but are time-consuming and expensive. Their role in routine staging before planning treatment must require clearer definition, whilst from the practical point of view, they seem to be of minor importance [39, 40].

### LN metastasis

Very early in the course of the disease, mesenteric LNs metastases are invariably involved as single or multiple metastases, regardless of the size of the primary tumours, and are verified in up to 88% along the superior mesenteric vessels [35, 41]. LN metastasis were documented in even 46.9% of patients with siNENs < 10 mm [42•].

Extra-abdominal LN metastases were observed in 24 (4.0%) of all patients at diagnosis and 37 (6.1%) patients displayed extra-abdominal metastases (other than metastatic LNs) at diagnosis [5••].

Whilst primary tumours are rarely visualised on CT, mesenteric LN metastases typically and often appear as contrast-enhancing soft-tissue masses with fibrotic bands radiating outward into the mesenteric fat in a stellate pattern surrounding LN metastasis [43•].

LN metastasis tends to develop a “desmoplastic stromal reaction”, which is the result of coordinated changes in several stromal cells under the control of a single gene product, the CD36 protein. The repression of CD36 leads to a decrease in fat accumulation and an increase in matrix deposition [43•].

Obliterative processes with considerable variation in the degree and rate of progression of “desmoplasia” may cause vascular encasement. Clinically, this may lead to abdominal pain, disabling diarrhoea, weight loss to the extent of malnutrition and eventually the risk of life-threatening situations with acute intestinal obstruction or intestinal gangrene.

The current TNM classification (Table 1) subdivides patients with pathological (p) N1 and pN2 LN involvement [44••].

In the current pathological LN classification stage, pN1 by definition includes those patients with less than 12 regional LNs affected without mesenteric mass(es) greater than 20 mm in size. Patients with 12 or more positive regional nodes and/or mesenteric mass(es) greater than 20 mm in maximum dimension are defined as pN2. The number of involved LNs is of prognostic relevance [45].

The number of resected and histopathologically examined and involved LNs and the LN ratios (involved nodes:total nodes) was prognostic for overall survival—patients with 12 or more resected and examined LNs had the best survival outcomes [46].

A lower metastatic node ratio predicted improved survival on multivariable analysis and is associated with high-volume institutions which are more experienced in LN dissection [42•].

The WHO classification is not helpful in clarifying the possibility of radical LN dissection, an important step in treatment decision-making. The neighbourhood of the LN bulk and possible vascular involvement are to be assessed by CT/magnetic resonance imaging (MRI) angiography (coronal and sagittal view).

Ohrvall et al. [47••] proposed a surgical “LN staging (=level) classification” by evaluating the operability of primary and mesenteric LN metastases. LN involvement level 1 consists of tumours located close to the intestine, whilstLN level 2 tumours involve arterial branches near their origin in the mesenteric artery. Level 3 LN tumours extend along (without encircling) the superior mesenteric artery trunk, and level 4 LN metastases extend retroperitoneally, behind or above the pancreas, or grow around the mesenteric artery and involve the origin of proximal jejunal arteries on the left side of the superior mesenteric artery.

In their series [47••], 24% of 56 patients were surgically staged LN level 1, 22% staged level 2, 28% staged level 3 and 16% staged level 4. Level 4 LN tumours involved more proximal parts of the mesenteric artery, sometimes growing circumferentially around this vessel, and were not removable.

These findings were confirmed by Lardière-Deguelte et al. [48] who retrospectively analysed morphological imaging, consisting of early arterial-phase contrast-enhanced abdominal-pelvic CT and/or MRI, in an attempt to develop criteria to prospectively predict the operability of mesenteric LNs.

A key issue in siNEN resection is not necessarily the primary tumour itself. Surgery is to focus on preserving bowel function whilst selectively resecting mesenteric LNs. LN metastases have to be removed by cautious dissection around the superior mesenteric vessels in order to preserve the vascular supply to the small intestine and thus to avoid overextensive bowel resections. LN dissection along the vessels may be technically challenging, especially in complex situations with level and LN involvement. An appropriate preoperative LN cartography with respect to the jejunal vascular collaterals along the superior mesenteric vessels could help to estimate the technical demand of surgery. Radical lymphadenectomy along the superior mesenteric vessels may not necessarily require extended small bowel resection, which may cause “short bowel syndrome” and compromise patients’ quality of life [48, 49••].

Skip metastases (LN metastases outside the “expected” lymphatic drainage) may occur in up to two thirds of patients, which may mandate more extensive lymphadenectomy to prevent unresectable locoregional recurrence [50].

Patients with local tumour-related symptoms generally undergo local resection at the time of diagnosis. However, some symptomatic patients have advanced level 4 LN metastases, encasing the superior mesenteric vessels and rendering radical resection challenging. In highly advanced situations including the infeasibility to remove the LN bulk with an acceptable level of morbidity, stenting and other palliative methods improve the clinical symptoms and prevent bowel ischaemia [51].

### Distant metastasis

The liver and the peritoneum are the two most common distant metastatic sites. With regard to the site of distant metastasis [52], 80/219 (36.5%) patients presented with hepatic metastasis only (M1a), 14 (6.39%) with peritoneal metastasis only (M1b), and 53 (24.2%) with both hepatic and peritoneal metastases (M1c) at the time of surgery or during follow-up, whilst 71 (32.4%) patients were classified as M0.

Solitary liver metastases are the exception. In only 1 of 40 patients, a solitary liver lesion with curative outcome after resection was documented [35].

The majority of liver metastasis is multiple and often less than 10 mm in diameter. Because of their typical bilobar growth pattern, many resections of hepatic metastatic NENs that are considered curative at the time of surgery are palliative [35].

At the time of initial diagnosis, fewer than five metastases in one lobe were revealed in 80 (21.9%) of 366 patients, 105 (28.7%) patients had bilobar and/or 5 to 10 metastases and 139 (38.0%) patients had more than 10 liver metastases, respectively. Ninety-nine (42%) patients without liver metastases at initial diagnosis developed liver metastases during follow-up [5••].

Morphologically, three different patterns of liver infiltration by metastasis are to be differentiated, since they have an impact on the therapeutic approach [19, 53•]: in the “simple pattern” ([A]; type 1), the metastases correspond to a single metastasis or to metastases confined to one liver lobe or are limited to two adjacent segments so that they can be treated with a standard anatomical resection. In the “complex pattern” ([B]; type 2), there is one major focus (metastatic bulk) with accompanying smaller deposits contralaterally. Diffuse, multifocally disseminated metastatic spread involves all parts of the liver ([C]; type 3). Analysing patients with siNENs and liver metastasis, 5 (14.3%) of 35 were classified as type 1, 8 (22.9%) as type 2, and 22 (62.9%) as type 3, respectively.

A bilobar pattern can still be approached surgically when 70 to 90% of the metastasis can be removed (cytoreduction/debulking) [53•, 54]. However, only up to 20% of patients may be candidates for surgery [53•].

Besides liver metastases in 48 (57.1%) of 84 patients, solely peritoneal and bone metastases were observed in 2 (2.4%) and 1 (1.2%) patients, respectively [41].

Ovarian metastases occur in 4% of patients (potentially causing carcinoid syndrome) followed by pancreatic (0.5%) and splenic metastases (0.5%) [5••].

The presence of carcinoid heart disease and mesenteric LN metastases, distant abdominal LN metastases, liver metastatic burden, extra-abdominal metastases, skeletal involvement and PC are independent prognostic factors for overall survival in siNENs [5••]. Bone metastases are prognostically relevant [55].

Although occurring in only approximately 5%, lung metastases may impact patients’ outcome. The development of metachronous lung metastasis is associated with concomitant disease progression in established abdominal metastasis in most patients [56].

### Staging of the disease

The specific oncological characteristics of siNENs emphasise the necessity of exact (T)NM staging prior to planning further treatment. This is the prerequisite before selecting any treatment regime.

In general, routine morphological radiological imaging techniques (abdominal US, CT, magnetic resonance tomography [MRT]) tend to significantly underestimate the neuroendocrine disease [57].

Preoperative morphological imaging “understaged” the disease in 14/20 (70%) when compared with intraoperative findings. In patients with multifocal primary tumours and miliary liver metastasis, no imaging modality was able to detect the entire extent of disease spread [41].

The currently preferred hybrid imaging method combines the advantage of CT or MR and SSA receptor imaging in demonstrating the anatomical/morphological details of a lesion and its functional characteristics. The further development of radioactively labelled derivatives of synthetic SSA and the improvement of positron emission tomography (PET) technology, with greater resolution and simultaneous specific binding of radiopharmaceuticals to various somatostatin (SS) receptor subtypes, have improved the staging of siNENs compared to SS receptor scintigraphy alone.

^68^Ga-DOTA-SSA PET/CT or MRT is recommended for functioning imaging in G1 and G2 tumours. This technique facilitates the diagnosis and extent of LN and distant metastases, such as peritoneal, liver and bone lesions [58].

Whole-body scans enable more exact staging and also the diagnosis of primaries in up to 89% of cases [59].

^18^FDG-PET/CT is better suited for G3 tumours, which generally have a higher level of glucose metabolism and less SS receptor expression than low-grade NENs, and additionally provides prognostic information.

However, single “hot spots” in functional imaging do not exclude multicentric disease. Therefore, if surgery is indicated, meticulous intraoperative abdominal examination with bidigital exploration of the small bowel, the liver and the abdominal cavity is to be performed as palpation is superior to any imaging technology [59].

Differences in preoperative hybrid imaging and intraoperative findings may influence the surgical strategy.

### Treatment of the primary siNEN and LN metastasis (N1/2) in patients with (M1) and without (M0) distant metastasis

Positive LNs and distant metastasis in preoperative imaging do not per se exclude patients from surgery if they are fit for such treatment (heart disease not present or treated efficiently), and the siNEN itself or the distant metastasis is graded as G1/G2 [60••].

Traditionally, the approach of choice is laparotomy with meticulous exploration of the entire abdominal cavity excluding or verifying small extrahepatic metastasis with bidigital small bowel palpation to exclude multifocality and to determine the exact anatomy of LN metastasis to the superior mesenteric vessels [61].

Laparoscopic procedures with the intention of curative surgery remain controversial [7•, 41, 62]. The level of evidence of the role of laparoscopic surgery for si-NENs is low. Laparoscopic techniques are feasible and safe and may be considered, provided that oncological surgical standards in treating siNENs are achieved. However, patients with high-grade mesenteric infiltration and multiple tumours do not seem to be optimal candidates for extended laparoscopic LN dissection.

In palliative intent, the laparoscopic approach seems to be advantageous for the resection of local disease in patients with unresectable LN and liver metastases [61].

### Arguments for locoregional surgery

Although primary siNENs may have an indolent clinical local course for a long time, radical locoregional surgery should be discussed in all patients with locoregional siNENs, with or without distant metastasis, who fulfil the basic criteria [6, 7•, 9•, 10•, 63, 64].

The aim of locoregional prophylactic surgery in M0 and M1 patients is to improve quality of life by averting abdominal complications caused by unpredictable progressive local tumour growth which may ultimately result in bowel obstruction with the consequence of emergency surgery. Growing LN metastasis with fibrosis around the main vessels may result in a state of chronic malnutrition and severe intestinal ischaemia. These life-threatening local complications can be avoided by LN dissection as long as only LN levels 1 to 3 are affected [24, 65••].

Depending on the location and number of the primaries, the optimal surgical treatment consists of sparing segmental small bowel or ileocaecal resection (for distal ileal tumours) with dissection of the regional LNs along the superior mesenteric vessels up to the inferior border of the pancreas (stage = levels 1 to 3 [4]).

#### M0

In patients with stage I to III disease, resection of the primary tumour and regional lymphadenectomy is indicated as a curative procedure which improves survival [66•].

In patients with radically removed positive LNs (pN1M0), the 1-, 5- and 10-year survival rates were 100%, 100% and 83 ± 15%, respectively [35].

The completeness of LN dissection also influences prognosis. Both overall survival and relative survival were significantly improved in the patients whose metastatic mesenteric LNs were grossly radically removed (R0) as compared to those with remaining pathological mesenteric LNs (R1) [5••].

After curative surgery, there is no indication for specific medical treatment, and there is no proven role for neoadjuvant or adjuvant medical treatment in si-NEN patients.

#### M1

Surgery is recommended in selected patients in the “simple (type 1) pattern” of liver involvement with the chance to cure locoregional disease alongside synchronously performed radical liver surgery.

In stage IV disease with complex (type 2 or 3) liver involvement or unresectable extrahepatic distant metastasis, locoregional surgery is indicated in locally symptomatic patients [8•].

Generally not considered to be curative, the value of locoregional surgery in asymptomatic patients is discussed controversially. After propensity score matching patients with and without prophylactic locoregional resection, Daskalakis et al. found no survival benefit comparing the two groups [67••].

The first systematic review of the literature described a clear trend towards improved survival after resection [14••]. Additional systemic reviews and meta-analyses [13••, 15••, 16••] support the results authored by Capurso et al., demonstrating that palliative resection of primary siNENs in the setting of unresectable metastatic disease may improve survival.

The results of all the reviews and meta-analyses must be interpreted with caution due to potential selection and publication bias. The data support the consideration of surgery, particularly in patients with low tumour load and good functional status.

Because large, randomised prospective studies investigating the management of primary tumour and regional LN metastasis in association with liver metastases are lacking, the optimal treatment strategy remains debatable and should be discussed individually in multidisciplinary tumour boards.

Resection of liver metastasis may improve survival [68, 69, 70, 71, 72, 73] with a 10-year survival rate of approx. 50 to 60% in patients having undergone either surgical resection or ablation of liver metastases. However, the cure rates are low and recurrence in the liver is frequent.

Factors influencing prognosis are unclear. Manguso et al. [74•] recently evaluated how the extent of liver resection (complete resection ([CR], partial resection [PR], or no resection [NR]) influences outcomes after complete resection of the primary tumour.

The 5‐year rate of overall survival was 79.4% for NR, 84.7% for PR and 100% for CR, demonstrating a trend that CR was the best, followed by PR then NR. However, 10‐year overall survival showed no significant differences (72.7% NR; 84.7% PR; 82.5% CR). More than 10 liver lesions or receiving chemotherapy were negative predictors of survival.

The minimal criteria required for liver surgery with “curative intent” are resectable G1/G2 liver disease with acceptable levels of morbidity (< 20%) and mortality (< 5%), the absence of right heart insufficiency and of extra-abdominal metastases (previously assessed by hybrid imaging), the absence of diffuse PC and a locoregionally resectable or already resected tumour.

Locoregional resection performed by an experienced surgical team following a standardised surgical technique with low morbidity and mortality [48] has the objective to make unresectable liver metastases the only persisting problem. The selective treatment of the liver by various ablative therapies may improve overall prognosis [18].

With regard to peritoneal metastases, surgical resection is recommended synchronously with the primary tumour, LN and hepatic metastasis, if feasible, to control symptoms [7•, 64].

## Conclusion

In stage I to III disease, locoregional surgery may be curative. In stage IV disease, surgery is recommended in selected patients with the chance to cure locoregional disease besides radical liver surgery in the “simple (type 1) pattern” of liver involvement. In stage IV disease with complex (type 2 or 3) liver involvement or unresectable extrahepatic distant metastasis, locoregional surgery is indicated in locally symptomatic patients. Resection of the primary tumour should also be attempted in locally asymptomatic patients as the overall outcome may be better in patients after primary tumour resection even with distant metastasis although a direct causal relationship has not been proven to date. The type of surgery should be individualised, and no general approach can be recommended. The risks and benefits of the surgical intervention and the merits of pharmacological treatment (e.g. SSAs) have to be balanced. As such, the treatment of such patients is driven primarily by surgeons’ experience, and available data are based predominantly on retrospective studies. Keeping this in mind, current recommendations advocate for patients with siNENs (localised or metastatic) to be managed by multidisciplinary teams in experienced centres.
